# Lateral femoral cutaneous nerve block with different volumes of Ropivacaine: a randomized trial in healthy volunteers

**DOI:** 10.1186/s12871-019-0833-4

**Published:** 2019-08-28

**Authors:** Frederik Vilhelmsen, Mariam Nersesjan, Jakob Hessel Andersen, Jakob Klim Danker, Leif Broeng, Daniel Hägi-Pedersen, Ole Mathiesen, Kasper Højgaard Thybo

**Affiliations:** 1 Department of Anesthesiology, Naestved Hospital, Naestved, Denmark; 2 CAR, Department of Anesthesiology, Centre of Anaesthesiological Research –ZealandUniversity Hospital, Koege, Denmark; 3 Department of Orthopedics, Zealand University Hospital, Koege, Denmark

**Keywords:** Regional anesthetics, Lateral femoral cutaneous nerve block, Total hip arthroplasty, Ropivacaine, Anatomy, Clinical trial

## Abstract

**Background:**

Nerve block of the lateral femoral cutaneous nerve (LFCN) is a predominantly sensory block. It reduces pain following total hip arthroplasty (THA), but the non-responder rate is high. We hypothesized, that an increased volume of ropivacaine, would result in greater coverage of incisions used for THA.

**Methods:**

We conducted a randomized, blinded trial in 20 healthy volunteers. Participants were randomized to receive bilateral LFCN-blocks with 8 mL ropivacaine 0.75% on the left side and 16 mL ropivacaine 0.75% on the right side, or vice versa. Allocation was blinded to both participants and outcome assessors. Before nerve block performance, incision lines for posterior and lateral THA approaches were depicted with invisible ultraviolet-paint, thereby securing sufficient blinding during outcome assessment. The blocked area was mapped using temperature and mechanical discrimination tests. Quadriceps muscle strength was monitored. Primary outcome was coverage of the posterior incision line assessed by temperature discrimination test.

**Results:**

We found no difference in coverage of the posterior or lateral incision lines when comparing LFCN-blocks with 8 mL versus 16 mL of ropivacaine. The blocked area was significantly larger in the 16 mL group, assessed by both temperature discrimination test (*p* = 0.012) and mechanical discrimination test (*p* = 0.034). We observed no difference between groups regarding quadriceps muscle strength (*p* = 1.0).

**Conclusions:**

A LFCN-block with increased volume of ropivacaine from 8 mL to 16 mL did not result in a greater coverage of posterior or lateral incision lines used for THA, but in a larger blocked sensory area.

**Trial registration:**

Clinicaltrials.gov: NCT03138668. Registered 3rd of May 2017.

## Background

Total hip arthroplasty (THA) is often associated with moderate to severe postoperative pain [1] and peripheral nerve blocks have been used as part of a multimodal analgesic strategy [2–5]. The use of peripheral nerve blocks may be hindered by an accompanying motor blockade, which may lead to increased risk of falling [6].

The lateral femoral cutaneous nerve (LFCN) block is a predominantly sensory block and has been suggested to mitigate pain after hip surgery [7–9]. A clinical trial from 2016 showed that LFCN-blocks with 8 mL ropivacaine 0.75% reduced moderate to severe movement related pain after THA surgery using the posterior approach, but the non-responder rate was high (42%) [8]. Recently, several other studies have investigated the anatomical sensory distribution of a LFCN-block and consistently revealed a limited coverage of THA incisions using 5 to 10 mL of local anesthetics [10–12].

The LFCN most commonly originates from L2 and L3 spinal nerves and travels across the iliac muscle towards the anterior superior iliac spine (ASIS) as one single nerve, before passing beneath the inguinal ligament (IL). In most cases it divides immediately into two branches distal to the IL and continues superficially to supply the lateral aspect of the thigh. However various branching patterns and points of exit from the pelvis have been reported. Variations include, but are not limited to, bifurcation or even trifurcation within the pelvis, exits point through the IL, lateral to the ASIS, or through the ASIS. [13–15]. The LFCN has recently been described as being placed in its own fascial canal in the thigh, named the LFCN-canal [16] or fat-filled flat tunnel [10], created by a splitting of the fascia latae forming a lumen in which the nerve lies.

We hypothesized that injection of a larger volume of local anesthetic could migrate both proximally and distally within the above-described canal, spreading to additional lesser, local cutaneous branches arising from LFCN, thereby giving rise to a greater blocked distribution area. In this trial, we investigate if LFCN-blocks with 16 mL ropivacaine results in a greater coverage of the incision line for the posterior THA approach compared with LFCN-blocks with 8 mL ropivacaine, assessed by temperature discrimination test (primary outcome).

## Methods

We conducted a randomized, blinded trial in healthy volunteers at the Department of Anesthesiology, Zealand University Hospital Koege, Denmark from the 19th of May 2017 to the 9th of June 2017.

### Participants

Inclusion criteria were: ≥ 18 years of age, American Society of Anesthesiologists physical status classification (ASA) score of I or II, Body Mass Index between 18 and 30. Exclusion criteria were inability to understand or speak Danish, inability to cooperate, allergies to ropivacaine, alcohol or drug abuse (investigators judgement), consumption of prescription-required analgesic drugs within four weeks before the trial date or over-the-counter analgesic drugs within 48 h of trial date, neuromuscular defects, former surgery in lower extremity, large tattoos or trauma on the thigh or hip, and diabetes mellitus. Women had to use hormonal contraception and give a negative urine Human Chorionic Gonadotropine test prior to inclusion.

Each participant was screened for the above criteria on the day of the trial and gave their written informed consent before trial inclusion.

### Incision lines

Before block performance, two hypothetical incisions were drawn on each side of the participants’ hips, corresponding to the posterior [17] and lateral [18] approaches for THA. It should be noted that the lateral approach for any practical conditions corresponds to the anterolateral approach with respect to skin incision. The lines were approximately 9–13 cm in length. This was done by author LB, who is an orthopedic surgeon specialized in THA surgery. The incisions were drawn with ultraviolet (UV) paint (SkinSafe UV12 blue reflection, J & Maya, Denmark), only visible when exposed to UV light. To ensure that only the orthopedic surgeon knew the exact placement of the incision lines, drawing of the incision lines took place behind a curtain and with blindfolding of the participants.

### Randomization and blinding

Participants were enrolled and allocated at random into two groups. Group A received a LFCN-block with 8 mL ropivacaine 0.75% on their right side and a LFCN-block containing 16 mL ropivacaine 0.75% on the left side. Group B received 16 mL ropivacaine 0.75% on the right side and 8 mL ropivacaine 0.75% on their left side.

The pharmacy of The Capital Region (Herlev, Denmark) prepared a computer-generated simple randomization list (1:1 ratio) of identification (ID) numbers and cognate allocation group, and packed the trial medicine in accordance, one medicine package for each ID number. The ID numbers were assigned consecutively. Each medicine package included two identical ampoules containing 20 mL ropivacaine 0.75%. Ampoules were labelled with respect to which volume to administrate to which side. Due to the volume difference, it was possible to visualize which side would be given the larger dose. The anesthesiologist (JKD) who administered the blocks could not be blinded and was therefore not further involved in the study. The preparation of the trial medicine was double-checked by a person not otherwise involved in the trial. To assure blinding of outcome assessors and participants, block performance took place behind a curtain and participants were blindfolded throughout the preparation and administration of the nerve blocks.

### Nerve block procedure

**Fig. 1:**
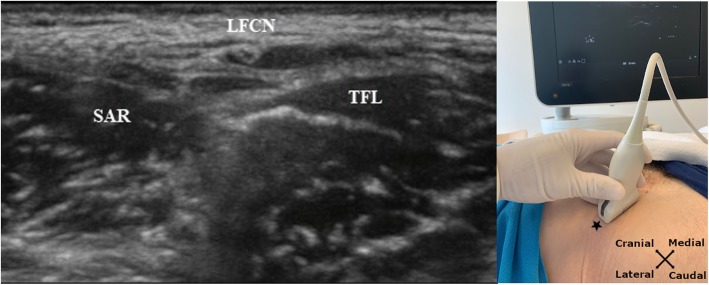
Ultrasound image of the target nerve. The image is not from an actual trial participant. SAR = sartorius muscle. LFCN = lateral femoral cutaneous nerve. TFL = tensor facia latae muscle. * marks the anterior superior iliac spine

Intravenous access, standard monitoring and baseline measurements were obtained before the block procedure. For the block procedure we used a Philips Sparq ultrasound system with a 4–12 MHz linear probe (Phillips, Netherlands) and a Pajunk SonoPlex 22G × 80 mm needle. With the patient in a supine position and the legs extended in neutral position, the transducer was placed parallel to the IL. With the femoral artery and vein as guidance the lateral part of the sartorius muscle and fascia latae was visualized. Approximately 3 cm inferior from this point, the branches of the lateral femoral cutaneous nerve was visualized in the hypoechoic fat-filled subfascial space between the sartorius muscle medially and the tensor fascia latae muscle laterally (Fig. 1). We used in plane approach and the needle was inserted at a shallow angle to reach the area of the nerve. The injection and spread of the local analgesics were visualized sonographically. The LFCN-block was performed on the right side first, immediately followed by the left side. Participants were monitored for 30 min after receiving the blocks. Failed blocks were predefined as preserved sensibility one hour after block performance when testing temperature discrimination in the examination area, described later.

### Outcomes assessment

Participants were introduced to all assessment measurements and baseline values were recorded prior to block procedure.

**Fig. 2:**
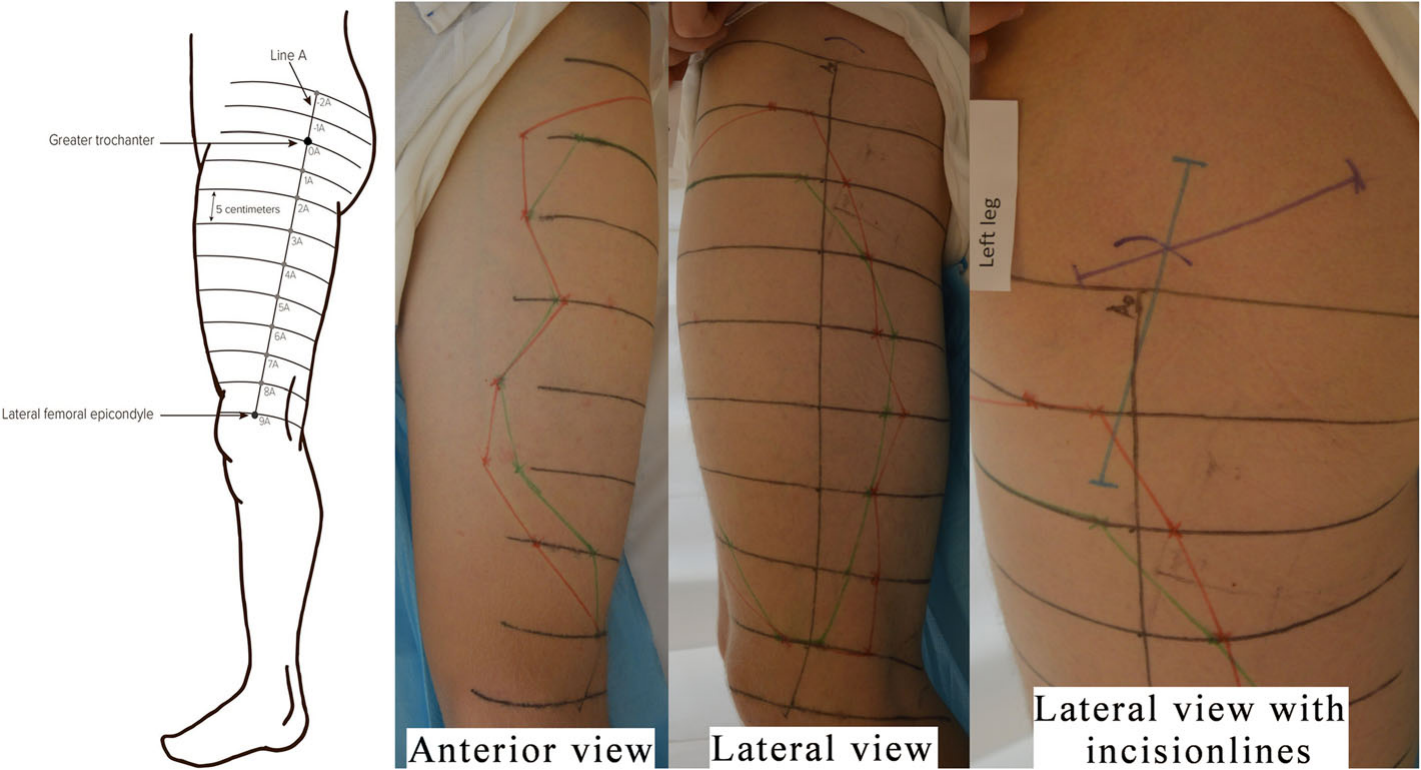
Mapping of blocked areas. Sketch showing how to draw guiding lines for the mapping of the blocked area, 0A marks the point of the greater trochanter. Red lines border the blocked area which was mapped using temperature discrimination test. Green lines border the blocked area which was mapped using pinprick. The purple line corresponds to the posterior incision line. The blue line corresponds to the lateral incision line. The drawn sketch has previously been published in “Sensory distribution of the lateral femoral cutaneous nerve block – a randomised, blinded trial.” Nersesjan M, Acta Anaesthesiologica Scandinavica volume 62 issue 6, Copyright© 2018, The Acta Anaesthesiologica Scandinavica Foundation. Published by John Wiley & Sons Ltd.

Assessment of outcome measures was done one-hour post-block to ensure full onset of the block. The affected area (blocked area) was assessed using two tests – a temperature discrimination test and a mechanical discrimination test (pinprick). Temperature discrimination is the ability to sense coldness when applying an alcohol soaked gauze to the skin, and pinprick is the ability to recognize sharp sensation when stimulating with a Von Frey filament (Somedic Senselab, size 19, pressure 137.3 g/mm^2^). To ensure a systematical exploration of the blocked areas, a line denoted “A” was drawn from the greater trochanter to the lateral epicondyle of the femur. This line was extended cranially. A line labelled “0A” was drawn perpendicular to line “A” at the point of the greater trochanter. Five cm above “0A” another line, also perpendicular to line “A”, was drawn and denoted “-1A”. Five cm beneath “0A” another line was drawn and denoted “1A”. Lines up until “15A” where drawn (Fig. 2). Testing with temperature discrimination test and pinprick along the above described lines, ensured a standardized way of measuring the blocked area on each leg of each participant. The blocked areas were marked and mapped with both measurements before the incision lines were visualized. The length and coverage of the incision lines for both temperature discrimination test and pinprick were recorded.

Furthermore, maximum pain during tonic heat stimulation (THS) was assessed using the Visual Analogue Scale (VAS) score (0–100 mm) (No pain: VAS = 0, worst pain imaginable: VAS = 100) to mimic the postoperative pain from incisions. This was done using a computer-controlled thermode (Modular Sensory Analyzer Thermal Stimulator, 2.5 cm^2^, Thermotest; Somedic A/B, Sweden) heated to 45^0^ C for 30 s. Two distinct measurements were recorded for each incision, one for the superior (cranial) part of the incision, and one for the inferior (caudal) part of the incision (baseline values were obtained by stimulating the skin superficial to the greater trochanter).

The maximal voluntary isometric contraction (MVIC) of the quadriceps muscle was measured with the participant sitting in an upright position, with their arms across the chest and a dynamometer in fixed position slightly above the ankle anteriorly. MVIC of the quadriceps femoris muscle were calculated in kilograms of force for each leg by the mean of three consecutive measurements. We predefined a decrease in MVIC to < 80% of baseline as clinically relevant.

### Outcome measures

All outcomes compared the sides given 8 mL ropivacaine with the sides given 16 mL ropivacaine. The primary outcome was: Difference in the percentage coverage of the posterior incision assessed by temperature discrimination test.

Secondary outcome measures were: Difference in percentage coverage of posterior incision assessed by pinprick (1 outcome); difference in the percentage coverage of the lateral incision assessed by both temperature discrimination test and pinprick (2 outcomes); difference in total blocked area assessed by temperature discrimination test and pinprick (2 outcomes); difference in number of patients feeling no pain (defined as VAS = 0 mm) assessed by maximum pain during THS test on the superior and inferior part of either incision lines (4 outcomes); and the difference in MVIC of the knee joint compared to baseline MVIC values (1 outcome).

### Statistical analysis

In a study from 2014 the mean percentage coverages of the incision lines were reported to be between 10.1–21.3% with a standard deviation (SD) of 15.7–24.5% [11]. We aimed to show an increase in coverage of the incision line from 20 to 40% by doubling the volume of ropivacaine injected from 8 mL to 16 mL. In a paired design with a type 1 error rate of 5%, a type 2 error rate of 10%, and an estimated SD of 25%, 18 participants were needed to show an increase in coverage from 20 to 40%. Taking the unknown true SD into account, we chose to enroll 20 participants.

Data were entered into two spreadsheets by two authors (FV and MN) and thereafter cross-checked for typos before further analysis. Using histograms, QQ-plots, and Shapiro-Wilk test, the outcomes regarding the incision lines showed non-normal distribution, whereas all other outcome measures showed normal distribution. We used Wilcoxon-Signed rank test and Hodges-Lehmann estimator for non-normally distributed data and paired t-test for normally distributed data. Chi-squared test and Fisher’s Exact test were used as appropriate (IBM SPSS statistics, release 24.0, IBM Corporation, Armonk, USA). The unblinding of the treatment allocation was done in two steps. First step unveiled the group allocation of participants. Calculations were completed, and conclusions drawn, before the final disclosure of the volumes administered in each group. A *p*-value of < 0.05 was considered statistically significant.

## Results

**Fig. 3:**
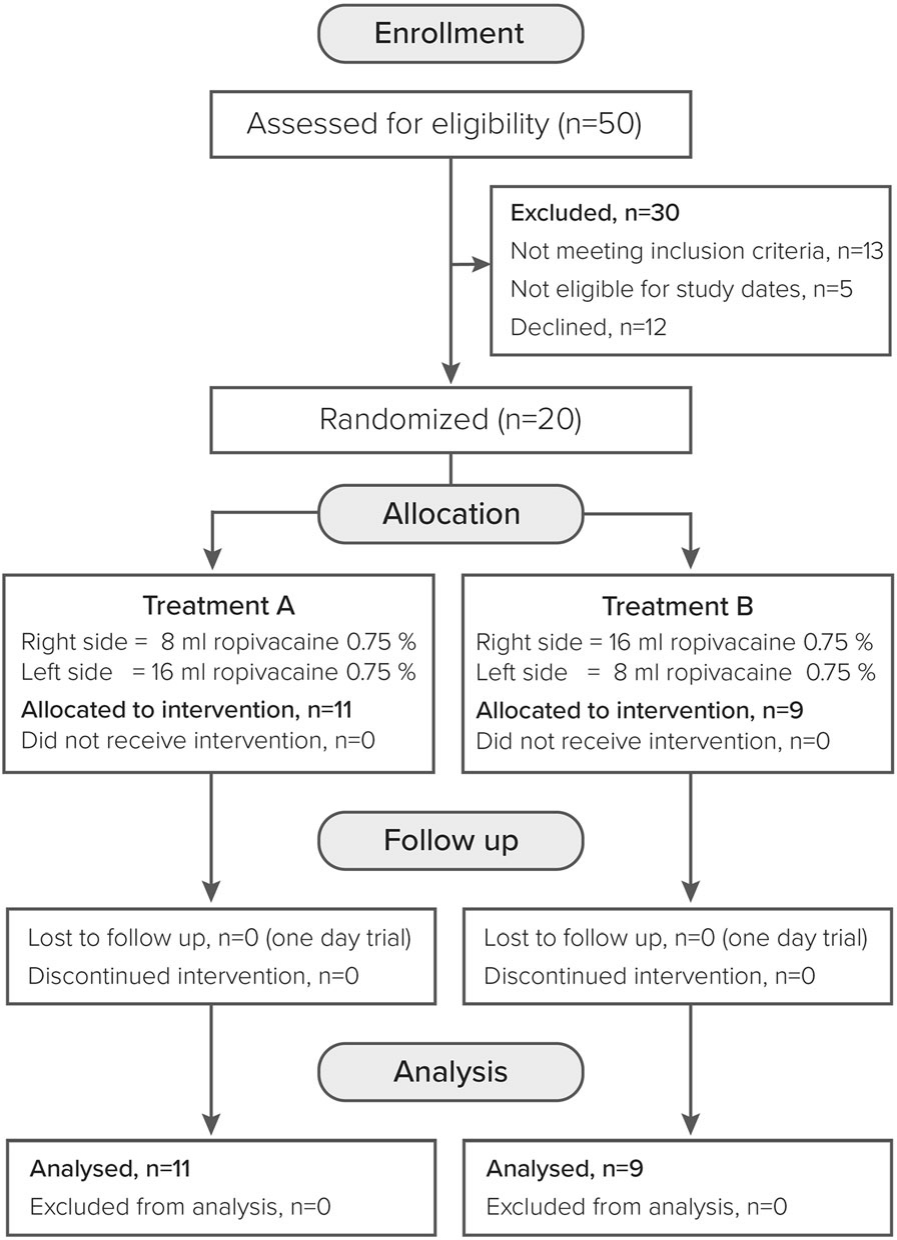
Consort flowchart

Fifty individuals were screened for eligibility, 30 were excluded and 20 were enrolled (Fig. 3). Eleven participants were allocated to treatment A and nine to treatment B. All enrolled participants received the intervention and completed the trial. Demographics and baseline values are shown in Table 1. We observed one failed block (2.5%). The median coverage of the posterior incision assessed by temperature discrimination test was 0.0% (IQR 0.0 to 0.0) in the 8 mL group and 0.0% (IQR 0.0 to 2.5) in the 16 mL group with a difference of 0.0% (95% CI: 0 to 3.5; *p* = 0.345). The other outcomes regarding the incision lines are shown in Table 2.

**Table 1:** Demographics and baseline values

Variable	Mean (Range)		
Age, [years]	25		(19–49)
Sex, male/female	13/7		
Height, [cm]	176		(164–186)
Weight, [kg]	70		(55–85)
Quadriceps femoris, MVIC, [kg]	Right leg Left leg	41 44	(26–58) (26–63)
Heat stimulation, VAS, [mm]	Right leg Left leg	41 44	(9–96) (11–94)

MVIC = Maximum Voluntary Isometric Contraction, VAS=Visual Analogue Score

**Table 2:** Outcomes

Variable	Group 8 mL		Group 16 mL		Difference and 95% CI		P
Coverage of posterior incision by temperature discrimination test, %	0.0	(0.0–0.0)	0.0	(0.0–0.0)	0.0	(0.0–3.5)	0.345
Coverage of lateral incision by temperature discrimination test, %	0.0	(0.0–19.3)	19.5	(0–45.3)	7.8	(−2.6–24.5)	0.221
Coverage of posterior incision by mechanical discrimination test, %	0.0	(0.0–0.0)	0.0	(0.0–0.0)	0.0	(0.0–0.0)	0.715
Coverage of lateral incision by mechanical discrimination test, %	0.0	(0.0–20.3)	0.0	(0.0–29.5)	3.9	(0.0–16.5)	0.110
Blocked area assessed by temperature discrimination test, cm^2^	418	(225.0)	564	(182.7)	146.3	(35.7–256.9)	0.012
Blocked area assessed by mechanical discrimination test, cm^2^	369	(211.4)	461	(156.0)	92.2	(7.8–176.6)	0.034
Post-block MVIC ≤80% of baseline, n	2		2				1.00
No pain during THS at superior portion of posterior incision, n	0		0				1.00
No pain during THS at superior portion of lateral incision, n	0		0				1.00
No pain during THS at inferior portion of posterior incision, n	0		1				1.00
No pain during THS at inferior portion of lateral incision, n	2		2				1.00

Coverage outcomes are reported as medians (IQR). Blocked areas are reported as means (SD). Maximal voluntary isometric contraction (MVIC) are reported as number of participants. Pain during tonic heat stimulation (THS) are reported as number of participants feeling no pain

The blocked sensory area was significantly larger in the 16 mL group compared with the 8 mL group when assessed by both temperature discrimination test and pinprick. The mean difference of the area assessed by temperature discrimination was 146.3 cm^2^ (95% CI: 35.7–256.9; *p* = 0.012), while the mean difference of the area assessed by pinprick was 92.2 cm^2^ (95% CI: 7.8–176.6; *p* = 0.034) (Table 2).

We observed a decrease in MVIC to less than 80% of baseline values in 2 of 20 legs receiving 8 mL ropivacaine and 2 of 20 legs receiving 16 mL ropivacaine (Table 2). None of the participants experienced bilateral decrease in MVIC to less than 80% of baseline.

No adverse events, both related or unrelated to the trial medication, were observed during the trial. Likewise, no participants reported any discomfort or paresthesia after the trial has ended.

## Discussion

This trial showed that using 16 mL 0.75% ropivacaine instead of 8 mL 0.75% ropivacaine for LFCN-block did not result in a larger coverage of the posterior or lateral incision lines typically used for THA [19, 20]. However, we observed a larger sensorial blocked area on the side receiving 16 mL compared with 8 mL ropivacaine. There was no difference in the number of participants with a decrease of 20% or more in MVIC of the quadriceps femoris muscle compared with baseline values.

The analgesic effect of nerve blocks after THA surgery is well established [5], however, the role of the LFCN-block in this setting is uncertain. As stated, an 8 mL ropivacaine 0.75% LFCN-block may relieve movement related pain among THA patients with moderate to severe pain to a certain degree, but with a relatively high non-responder rate [8]. We found no increase in coverage of the incision lines using 16 mL compared with 8 mL in this clinical model of healthy volunteers. Based on these results, we find it unlikely that a larger volume would lower the non-responder rate in the setting of THA surgery using the investigated approaches. It is theoretically possible that minimally invasive THA procedures utilizing smaller incisions could benefit more, but this has yet to be investigated.

**Fig. 4:**
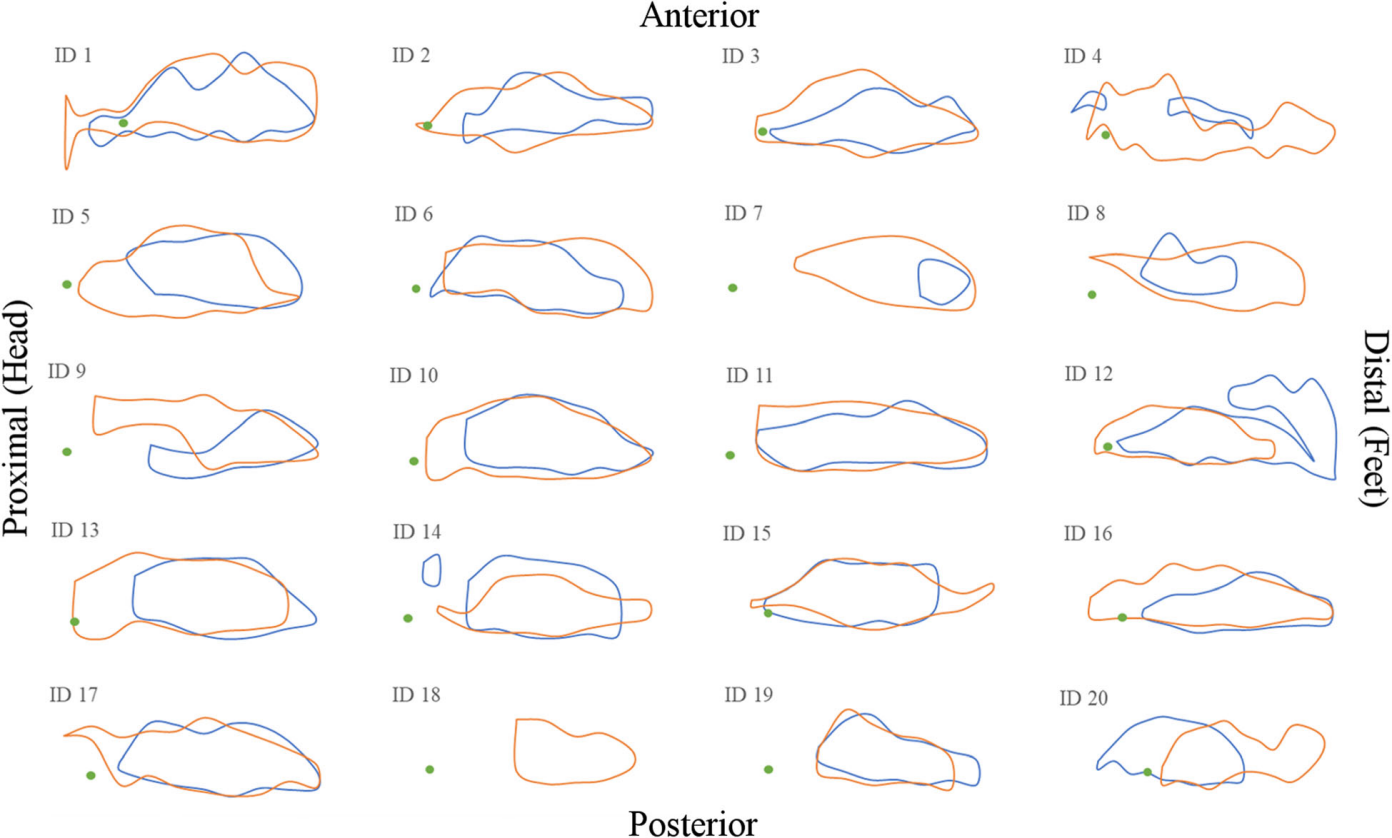
Area dot-plots. Blocked areas assessed with temperature discrimination for each participant, identification number given. The green dot marks 0A (the point of the greater trochanter). Blue lines represent the side given 8 mL. Orange lines represent the side given 16 mL

Injecting a larger volume of local anesthetics into the LFCN canal, however, did result in a larger blocked area, which may indicate the involvement of additional nerve branches. When dot plotting the blocked areas (Fig. 4) we saw that the blocks affected a generally larger area without spreading in any particular direction of the thigh. As hypothesized by Nielsen and colleagues [10] a larger volume injected in the LFCN-canal could spread to the proximal branch of LFCN. Our trial, however, did not confirm this hypothesis.

The increase in blocked area was approximately 30% and with no association between decrease in MVIC and a larger blocked area. The increase in area did not result in a better coverage of any of the tested incision lines for THA. Other types of surgery, as e.g., femoral fractures or plastic surgery procedures on the thigh may theoretically benefit from this larger blocked area.

This trial has the strengths of being blinded (participants, outcome assessors, and during statistical analyses) and designed with a bilateral setup, thereby limiting bias caused by anatomical variation between individuals. Furthermore, this trial (with respect to the 8 mL group) has the strength of reproducing the findings of Nersesjan and co-workers [12], which used the same methodology regarding block procedure and outcome assessment.

There are several limitations to this trial. First, the block technique used may not be ideal to block all branches of the LFCN, especially branches that cross posterior to the ASIS. A different technique (e.g. a more proximal approach to the LFCN) may yield a better coverage of the incision lines, or a larger area blocked.

Secondly, the incision lines drawn by the experienced orthopedic surgeon in this trial may not resemble the incision lines used by other surgeons. The surgeon was instructed to draw the incision lines as he would have if he were to perform THA surgery. Incision lines may vary between surgeons due to different patient characteristics and experience, or preferences, of the surgeon. Furthermore, they may not exactly match examples in textbooks.

Thirdly, the spread of the local anesthetic and the occurrence of failed blocks may vary between patients who have undergone THA surgery and healthy volunteers. This could be due to demographical differences, e.g. Body Mass Index, the surgical trauma, and, perhaps, the indication for THA in the first place (femoral neck fracture, osteoarthritis, osteonecrosis, etc.). This trial had all nerve block administered by an anesthesiologist who specializes in peripheral nerve blocks, which could explain the low failed block rate (2.5%).

Fourthly, the dose of ropivacaine was rather high in the 16 mL group (120 mg), especially if the LFCN-block were to be combined with other blocks. To resemble a clinical situation, a 16 mL diluted ropivacaine solution (i.e. 3.75 mg/mL) could have been used.

Lastly, this trial assumed that there were no intra-individual anatomical differences between the participants’ right and left sides. This was not further investigated and therefore might have influenced the results.

Further research should focus on combining LFCN with other nerve block techniques (e.g. the transmuscular quadratus lumborum-block [21–23], posterior femoral cutaneous nerve block, or an iliohypogastric block) to obtain a better coverage of the posterior incision lines after THA, or clinical trials investigating the analgesic effects of LFCN-block in THA patients using the lateral approach.

## Conclusions

In conclusion, a LFCN-block with 16 mL ropivacaine 0.75% did not result in a larger coverage of the posterior incision used for THA compared with a LFCN-block consisting of 8 mL ropivacaine 0.75%. It did, however, increase the blocked area. Based on results from this trial we cannot recommend increasing the volume of ropivacaine used in LFCN-blocks to achieve a larger coverage of incisions used in THA.

## Data Availability

Anonymized datasets from trial is available from the corresponding author on reasonable request.

## Abbreviations

ASA: American Society of Anesthesiologists physical status classification
ASIS: Anterior superior iliac spine
CI: Confidence interval
GCP: Good clinical practice
ID: Identification
IL: Inguinal ligament
IQR: Interquartile range
LFCN: Lateral femoral cutaneous nerve
mg: Milligrams
mL: Milliliters
MVIC: Maximal voluntary isometric contraction
SAR: Sartorius
SD: Standard deviation
TFL: Tensor fascia latae
THA: Total hip arthroplasty
THS: Tonic heat stimulation
UV: Ultraviolet
VAS: Visual analogue scale

## References

[1] Gerbershagen HJ, Aduckathil S, van Wijck AJ, Peelen LM, Kalkman CJ, Meissner W (2013). Pain intensity on the first day after surgery: a prospective cohort study comparing 179 surgical procedures. Anesthesiology.

[2] Kehlet H, Dahl JB (2003). Anaesthesia, surgery, and challenges in postoperative recovery. Lancet.

[3] Hojer Karlsen AP, Geisler A, Petersen PL, Mathiesen O, Dahl JB (2015). Postoperative pain treatment after total hip arthroplasty: a systematic review. Pain..

[4] Fischer HB, Simanski CJ (2005). A procedure-specific systematic review and consensus recommendations for analgesia after total hip replacement. Anaesthesia..

[5] Guay J, Johnson RL, Kopp S (2017). Nerve blocks or no nerve blocks for pain control after elective hip replacement (arthroplasty) surgery in adults. The Cochrane database of systematic reviews.

[6] Johnson RL, Kopp SL, Hebl JR, Erwin PJ, Mantilla CB (2013). Falls and major orthopaedic surgery with peripheral nerve blockade: a systematic review and meta-analysis. Br J Anaesth.

[7] Coad NR (1991). Post-operative analgesia following femoral-neck surgery--a comparison between 3 in 1 femoral nerve block and lateral cutaneous nerve block. Eur J Anaesthesiol.

[8] Thybo KH, Mathiesen O, Dahl JB, Schmidt H, Hagi-Pedersen D (2016). Lateral femoral cutaneous nerve block after total hip arthroplasty: a randomised trial. Acta Anaesthesiol Scand.

[9] Jones SF, White A (1985). Analgesia following femoral neck surgery. Lateral cutaneous nerve block as an alternative to narcotics in the elderly. Anaesthesia.

[10] Nielsen TD, Moriggl B, Barckman J, Kolsen-Petersen JA, Soballe K, Borglum J, et al. The lateral femoral cutaneous nerve: description of the sensory territory and a novel ultrasound-guided nerve block technique. Reg Anesth Pain Med. 2018. 10.1097/AAP.0000000000000737 29381568

[11] Davies A, Crossley A, Harper M, O'Loughlin E (2014). Lateral cutaneous femoral nerve blockade-limited skin incision coverage in hip arthroplasty. Anaesth Intensive Care.

[12] Nersesjan M, Hägi-Pedersen D, Andersen JH, Mathiesen O, Dahl JB, Broeng L, et al. Sensory distribution of the lateral femoral cutaneous nerve block – a randomised, blinded trial. Acta Anaesthesiol Scand. 2018. 10.1111/aas.13091 29468642

[13] Majkrzak A, Johnston J, Kacey D, Zeller J (2010). Variability of the lateral femoral cutaneous nerve: an anatomic basis for planning safe surgical approaches. Clinical anatomy.

[14] Tomaszewski KA, Popieluszko P, Henry BM, Roy J, Sanna B, Kijek MR (2016). The surgical anatomy of the lateral femoral cutaneous nerve in the inguinal region: a meta-analysis. Hernia.

[15] Corujo A, Franco CD, Williams JM (2012). The sensory territory of the lateral cutaneous nerve of the thigh as determined by anatomic dissections and ultrasound-guided blocks. Reg Anesth Pain Med.

[16] Hanna A (2017). The lateral femoral cutaneous nerve canal. J Neurosurg.

[17] Hoppenfeld S, deBoer P, Buckley R (2009). Surgical exposures in Orthopaedics : the anatomic approach. 4 ed.

[18] Mulliken BD, Rorabeck CH, Bourne RB, Nayak N (1998). A modified direct lateral approach in total hip arthroplasty: a comprehensive review. J Arthroplast.

[19] Chechik O, Khashan M, Lador R, Salai M, Amar E (2013). Surgical approach and prosthesis fixation in hip arthroplasty world wide. Arch Orthop Trauma Surg.

[20] Havelin LI, Fenstad AM, Salomonsson R, Mehnert F, Furnes O, Overgaard S (2009). The Nordic arthroplasty register association: a unique collaboration between 3 national hip arthroplasty registries with 280,201 THRs. Acta Orthop.

[21] Hockett MM, Hembrador S, Lee A (2016). Continuous Quadratus Lumborum block for postoperative pain in Total hip arthroplasty: a case report. A & A case reports..

[22] Johnston DF, Sondekoppam RV (2016). Continuous quadratus lumborum block analgesia for total hip arthroplasty revision. J Clin Anesth.

[23] La Colla L, Ben-David B, Merman R (2017). Quadratus Lumborum block as an alternative to lumbar plexus block for hip surgery: a report of 2 cases. A & A case reports.

[24] World Medical Association (2013). Declaration of Helsinki: ethical principles for medical research involving human subjects. Jama.

[25] ICH harmonized tripartite guideline (2001). Guideline for good clinical practice. J Postgrad Med.

